# Differences in Immunoglobulin Light Chain Species Found in Urinary Exosomes in Light Chain Amyloidosis (AL)

**DOI:** 10.1371/journal.pone.0038061

**Published:** 2012-06-18

**Authors:** Marina Ramirez-Alvarado, Christopher J. Ward, Bing Q. Huang, Xun Gong, Marie C. Hogan, Benjamin J. Madden, M. Cristine Charlesworth, Nelson Leung

**Affiliations:** 1 Department of Biochemistry and Molecular Biology, College of Medicine, Mayo Clinic, Rochester, Minnesota, United States of America; 2 Division of Nephrology and Hypertension, College of Medicine, Mayo Clinic, Rochester, Minnesota, United States of America; 3 Division of Gastroenterology and Hepatology, College of Medicine, Mayo Clinic Rochester, Minnesota, United States of America; 4 Department of Physiology and Biomedical Engineering, College of Medicine, Mayo Clinic, Rochester, Minnesota, United States of America; 5 Mayo Proteomics Research Center, College of Medicine, Mayo Clinic, Rochester, Minnesota, United States of America; University of Arkansas for Medical Sciences, United States of America

## Abstract

Renal involvement is a frequent consequence of plasma cell dyscrasias. The most common entities are light chain amyloidosis, monoclonal immunoglobulin deposition disease and myeloma cast nephropathy. Despite a common origin, each condition has its own unique histologic and pathophysiologic characteristic which requires a renal biopsy to distinguish. Recent studies have shown urinary exosomes containing kidney-derived membrane and cytosolic proteins that can be used to probe the proteomics of the entire urinary system from the glomerulus to the bladder. In this study, we analyzed urine exosomes to determine the differences between exosomes from patients with light chain amyloidosis, multiple myeloma, monoclonal gammopathy of undetermined significance, and non-paraproteinemia related kidney disease controls. In patients with light chain amyloidosis, multiple myeloma and monoclonal gammopathy of undetermined significance, immunoreactive proteins corresponding to monomeric light chains were found in exosomes by western blot. In all of the amyloidosis samples with active disease, high molecular weight immunoreactive species corresponding to a decamer were found which were not found in exosomes from the other diseases or in amyloidosis exosomes from patients in remission. Few or no light chains monomeric bands were found in non-paraproteinemia related kidney disease controls. Our results showed that urinary exosomes may have tremendous potential in furthering our understanding of the pathophysiology and diagnosis of plasma cell dyscrasia related kidney diseases.

## Introduction

Immunoglobulin light chain (AL) amyloidosis is the consequence of a plasma cell dyscrasia characterized by deposition of amyloid fibrils composed of immunoglobulin light chain [1]. For reasons incompletely understood, a small number of light chains misfold and form protofilaments. The protofilaments then assemble to form amyloid fibrils [2]. The processes of amyloid formation and deposition are toxic to organs resulting in progressive organ failure and eventual death if left untreated [3]. Since AL amyloidosis is dependent on the light chains, multiple myeloma is not required for its development although ∼20% of AL amyloidosis cases will have >20% plasma cells in the bone marrow. Because the source of amyloid formation is the monoclonal light chains, current treatments have focused on reducing the plasma cell population [4].

Besides AL amyloidosis, the kidney can also be affected by other plasma cell dyscrasias [5]. The most common diagnosis are monoclonal immunoglobulin deposition disease (MIDD) and myeloma cast nephropathy [6]. As in AL amyloidosis, the properties of the monoclonal light chains and not the plasma cell mass determine the kidney disease [7]. Therefore, the hematologic parameters such as monoclonal (M) protein concentration and bone marrow plasma cells percentage are not helpful in determining the renal diagnosis. Even proteinuria and urine M-protein spike may not accurately separate these diseases [8]. A renal biopsy is the only certain method of making the diagnosis.

Urinary exosomes are small extracellular vesicles (∼40–100 nm in diameter) that originate from all renal epithelial cells including glomerular podocytes, renal tubule cells and the cells lining the ureter and bladder [9]. Exosomes are formed as part of the multivesicular body (MVB) pathway in which intraluminal vesicles (ILVs) progressively accumulate during endosome maturation. They are formed by inward budding and scission of vesicles from the limiting endosomal membranes [10]. Exosomes are released from the MVB lumen into the extracellular environment during exocytosis. During this process, certain cytosolic proteins are incorporated into the invaginating membrane and engulfed in these vesicles, thereby maintaining the same topological orientation as the plasma membrane. Exosomes are thought to be involved with the removal of unwanted proteins and as acellular vehicles to transfer molecules among cells in normal and pathologic states (e.g., HIV) [11], although the exact role of urinary exosomes has not been elucidated yet. Numerous reports have shown amyloidogenic precursors associated with exosomes. Proteins associated with neurodegenerative disorders such as the prion protein in transmission spongiform encephalopathies, Amyloid Precursor Protein (APP) in Alzheimer’s disease, and mutations of cytosolic CuZn superoxide dismutase (SOD1) involved in the familial amyotrophic lateral sclerosis (ALS) can be incorporated into ILVs and released into the exosome-enriched extracellular environment [10]–[14].

Urinary exosomes are rapidly becoming a powerful tool in the study of renal disease. The fact that urinary exosomes are excreted from every renal epithelial cells (from the glomerular podocytes to the urinary epithelial cells lining the urinary drainage system) provides us with an opportunity to study proteins once were either difficult or impossible to reach [9], [15]. Already, proteomics studies are looking into ways of using urinary exosome to diagnose genetic diseases and characterize disease biomarkers [16]–[19]. Given exosomes’ unique insight into the intracellular environment, we undertook this study to evaluate the possible differences that we may observe among urinary exosomes from patients with different plasma cells dyscrasias. Our goal is to assess the use of urinary exosomes as a non-invasive, diagnostic tool for plasma cell dyscrasias that will offer a snapshot of what is occurring in kidney tissue.

## Methods

### Ethics Statement

Urine samples from patients with plasma cell dyscrasias were collected. Urine came from the Myeloma Amyloid Dysproteinemia Disease Oriented Group urine bank that gathers waste urine samples from patients who have given written consent for research use of their specimens. Approval from the Mayo Institutional Review Board was obtained in adherence with the Declaration of Helsinki.

The urine samples were selected based on each patient’s diagnosis. Because these were waste urine samples, the Mayo Institutional Review board decided that no further/additional consent was needed from the patients (after the patients signed the initial consent form). These samples were analyzed anonymously. This study was approved as a minimal risk study by the Mayo Clinic Institutional Review Board.

### Exosome Extraction and Fractionation

Exosomes were extracted and fractionated from urine collected and processed as reported previously [17], [20]. Briefly, 200 mL of urine were dialyzed against distilled deionized water using a dialysis membrane with 10,000 Da Molecular weight cutoff. The urine was then filtered using 0.2 µm membrane and a Complete® EDTA-free protease inhibitor tablet and 0.02% NaN_3_ were added before the sample was stored at 4°C until it was processed the next day. The sample was then centrifuged at 45,000 rpm in a T-647.5 rotor for 2 hours at 4°C. The glassy pellet was then resuspended in 0.25 mL of 0.25 M sucrose in 20 mM HEPES, pH 7.5 with Complete® EDTA-free protease inhibitor. The suspension was sonicated in a cup-horned sonicator (550 sonic dismembranator) for 15 seconds. Crude exosome samples were processed for further exosome fractionation using a D_2_O sucrose gradient [17]. Two 5–30% sucrose Deuterium oxide gradients were prepared and overlaid with 0.125 mL of crude exosome preparation and then centrifuged at 40,000 rpm for 24 hours. 6 mm fractions were removed from the gradient using a Biocomp ® Gradient Station (Biocomp, Canada), the fractions were collected and aliquoted. A portion of the fractions was stored at 4°C for immediate analysis while most of the sample was stored at –80°C.

### Western Blotting

We blotted the fresh fractions using the sheep free kappa or lambda light chain antibodies (1∶500) from The Binding Site, Inc. (San Diego, CA). For polycystin 1, a mouse monoclonal antibody generated in the Mayo Polycystic Kidney Disease laboratory, antibody7e12 (anti-LRR PC1) (1∶500) was used. Glomerular exosomes were identified with the rabbit anti-podocin antibody (1∶2000) from Sigma (St. Louis, MO). Intact immunoglobulins (IgG) were identified using the polyclonal anti human-IgG-HRP antibody from the SPIFE IgG IEF kit (Helena Laboratories, Beaumont, Texas).

### Immuno-gold Electron Microscopy

Immuno-gold electron microscopy was performed as previously reported [21]. Freshly prepared exosome specimens were loaded on 300 mesh copper formvar/carbon grids and incubated with sheep anti human kappa or lambda free light chain antibody from The Binding Site, Inc (1/10 and 1/20 dilution) in phosphate buffered saline at 4°C overnight. The grids were then incubated with a 10 nm anti-sheep immunoglobulin G gold secondary antibody (1/30 dilution) for 2 hours at room temperature. After washing, the specimens were further fixed in 1% glutaraldehyde/PBS, and stained and embedded with 2% methylcellulose solution containing 0.4% uranyl acetate for 5 min. Specimens then air dried and were imaged with a JEOL 1400 transmission electron microscope operating at 80 kV.

**Table 1:** Description of the patients involved in this study.

Patient	Age	Sex	Disease	M-protein	sFLC (mg/dL)	Proteinuria (g/d)
AL-ex1	74	M	AL	IgGλ+λ	13.0	9.7
AL-ex2	51	F	AL	IgGλ	8.67	1.0
AL-ex3	61	M	AL	λ	1.19	1.7
AL-ex4	66	M	AL	IgGλ	2.85	4.2
AL-ex5	53	M	AL	IgMκ	4.36	8.3
AL-ex6	48	M	AL	λ	19.2	5.2
AL-ex7	61	F	AL	λ	3.19	8.4
AL-ex8*	48	F	AL	κ	1.78	0.5
MM-ex1	32	M	MM	IgGκ	1.41	0.03
MM-ex2	66	M	MM	IgAκ	53.5	0.5
MM-ex3	65	M	MM	κ	105	0.4
MM-ex4	61	M	MM	IgGλ	48.3	0.3
MM-ex5	41	F	MM + cast nephropathy	IgGλ+λ	758	8.9
C-ex1	57	F	Membranous glomerulonephritis	None	n/a	0.8
C-ex2	54	F	MGUS	IgGλ+λ	4.53	2.8
C-ex3	28	M	IgA nephropathy	None	n/a	1.0
C-ex4	42	M	MGUS	λ	445	0.1
C-ex5	42	F	Normal	none	n/a	n/a

AL =  light chain amyloidosis; MM =  multiple myeloma; MGUS = monoclonal gammopathy of undetermined significance; M-protein =  monoclonal protein; sFLC = serum free light chain concentration in milligrams per deciliter. Proteinuria is the concentration of protein in urine in grams per day. * This patient in currently in complete hematologic and near complete renal response after autologous stem cell transplantation.

### Proteomics Analysis

The SDS-PAGE gel bands are prepared for mass spectrometry analysis using the following procedures. Silver stained gel bands are destained with 15 mM potassium ferricyanide and 50 mM sodium thiosulfate in water until clear, then rinsed with water several times to remove all color [22]. The bands are reduced with 30 mM DTT/50 mM Tris, pH 8.1 at 55°C for 40 minutes and alkylated with 40 mM iodoacetamide at room temperature for 40 minutes in the dark. Proteins are digested in-situ with 30 µL (0.004 µg/L) trypsin (Promega Corporation, Madison WI) in 20 mM Tris pH 8.1/0.0002% Zwittergent 3–16, at 37°C overnight, followed by peptide extraction with 40 µL of 2% trifluoroacetic acid, then 60 µL of acetonitrile. The pooled extracts are concentrated to less than 5 µL on a SpeedVac spinning concentrator (Savant Instruments, Holbrook NY) and then brought up in 0.15% formic acid/0.05% trifluoroacetic acid for protein identification by nano-flow liquid chromatography electrospray tandem mass spectrometry (nanoLC-ESI-MS/MS) using a ThermoFinnigan LTQ Orbitrap Hybrid Mass Spectrometer (ThermoElectron Bremen, Germany) coupled to an Eksigent nanoLC-2D HPLC system (Eksigent, Dublin, CA). The digest peptide mixture is loaded onto a 250 nL OPTI-PAK trap (Optimize Technologies, Oregon City, OR) custom packed with Michrom Magic C8 solid phase (Michrom Bioresources, Auburn, CA). Chromatography is performed using 0.2% formic acid in both the A solvent (98%water/2%acetonitrile) and B solvent (80% acetonitrile/10% isopropanol/10% water), and running a 5%B to 45%B gradient over 60 minutes at 350 nL/min through a Michrom Magic C18 (75 µm×150 mm) packed tip capillary column. The LTQ Orbitrap mass spectrometer experiment is set to perform a FT full scan from 375–1600 m/z with resolution set at 60,000 (at 400 m/z), followed by linear ion trap MS/MS scans on the top four [M+2H] ^2+^ or [M+3H] ^3+^ ions. Dynamic exclusion is set to 2 repeats of the same ion which is then placed on an exclusion list for 20 seconds. The lock-mass option is enabled for the FT full scans using the ambient air polydimethylcyclosiloxane (PCM) ion of m/z = 355.069933 for real time internal calibration giving <2 ppm mass tolerances of the precursor masses [23].

**Figure 1:**
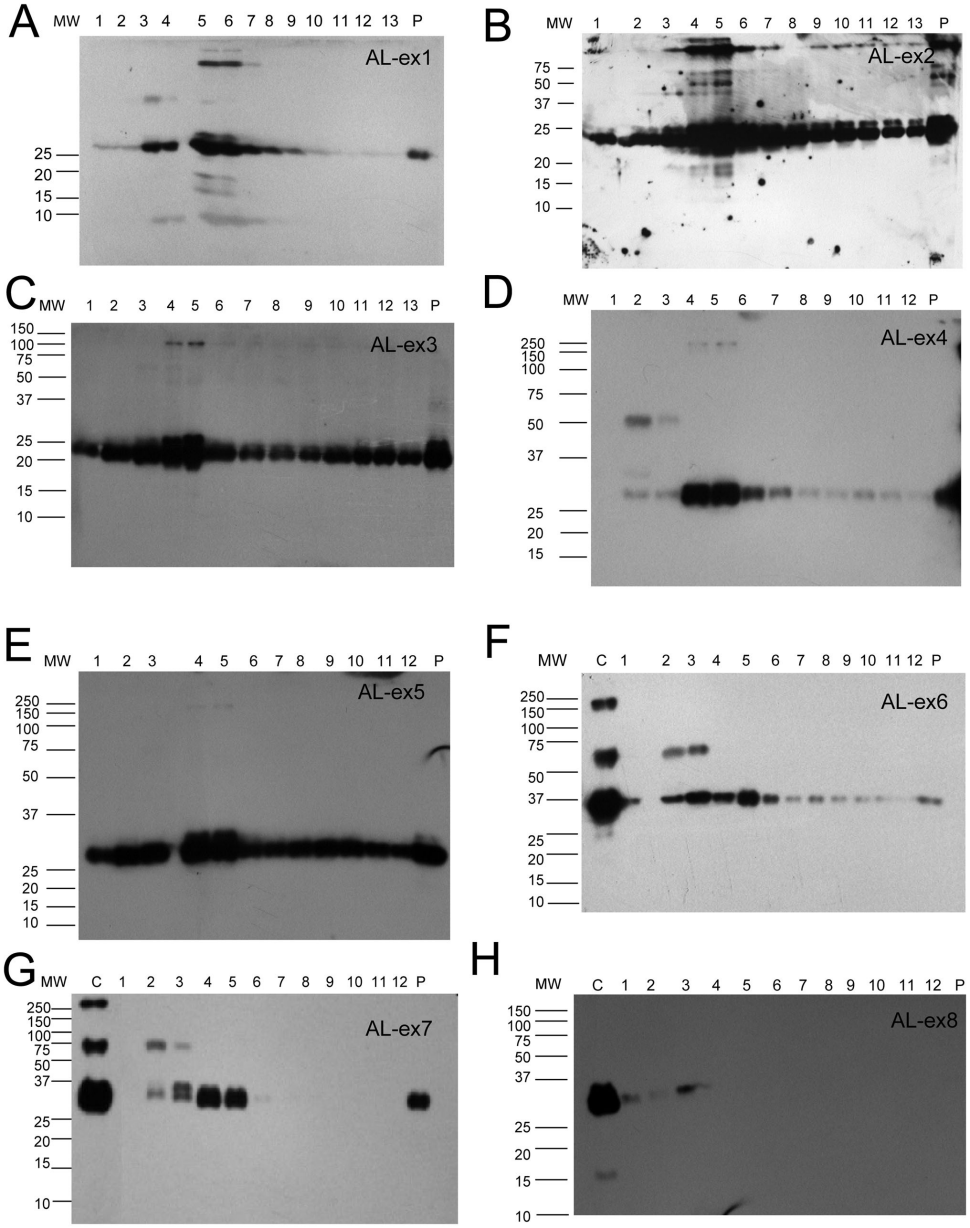
Western blot showing free light chain immuno-reactive proteins for AL patients. 15% SDS-PAGE gels under reducing conditions were used. The monoclonal proteins found in each patient are as follows: AL-ex1 = IgGλ + λ; AL-ex2 =  IgGλ; AL-ex3 = λ; AL-ex4 = IgGλ; AL-ex5 = IgMκ; AL-ex6 = λ; AL-ex7 = λ; AL-ex8 = κ.

**Figure 2:**
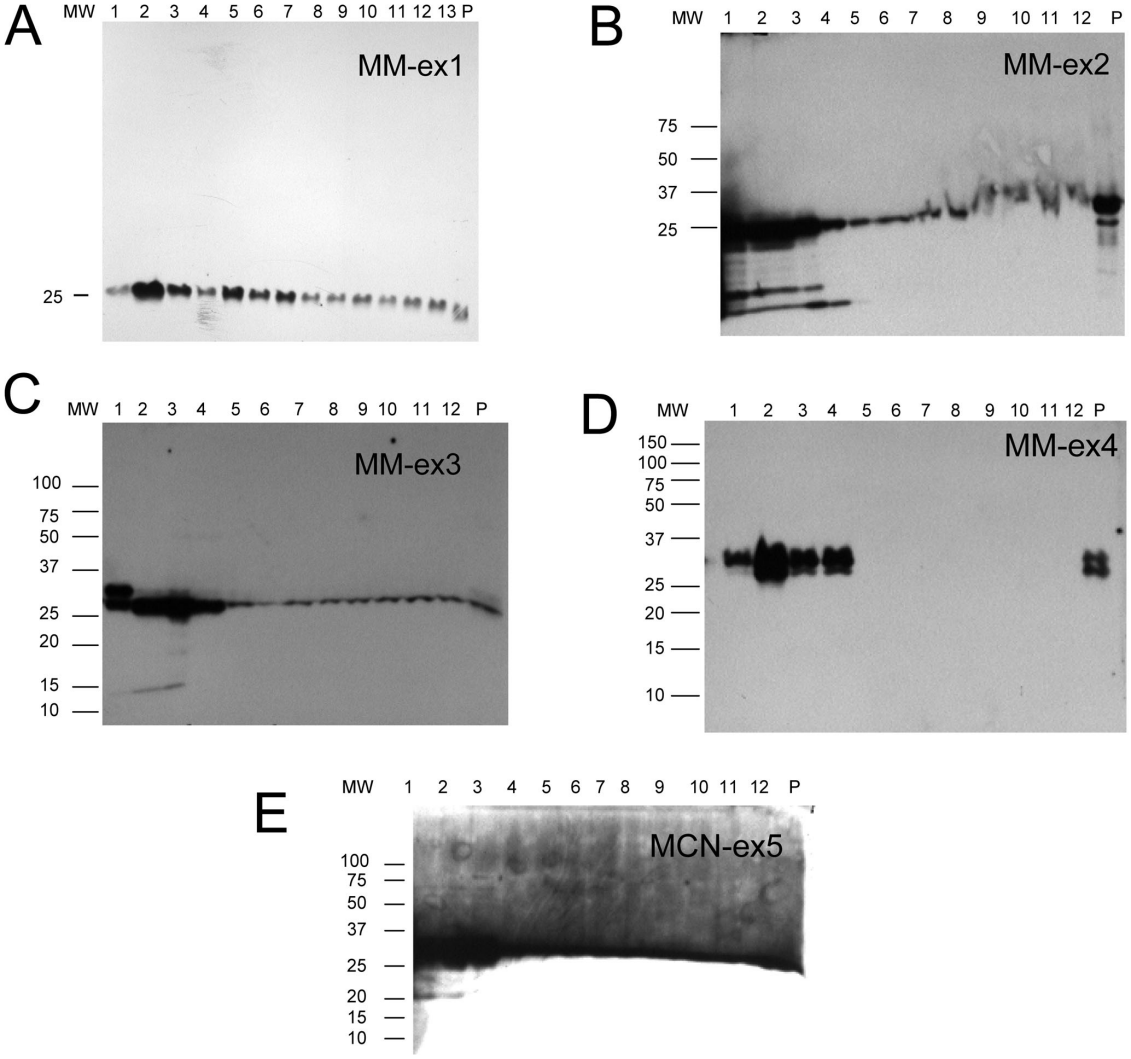
Western blot showing free light chain immuno-reactive proteins for MM patients. 15% SDS-PAGE gels under reducing conditions were used. The monoclonal proteins found in each patient are as follows: MM-ex1 = IgGκ; MM-ex2 = IgAκ; MM-ex3 = κ; MM-ex4 = IgGλ; MM-ex5 (also called MCN-ex5) = IgGλ+λ.

**Figure 3:**
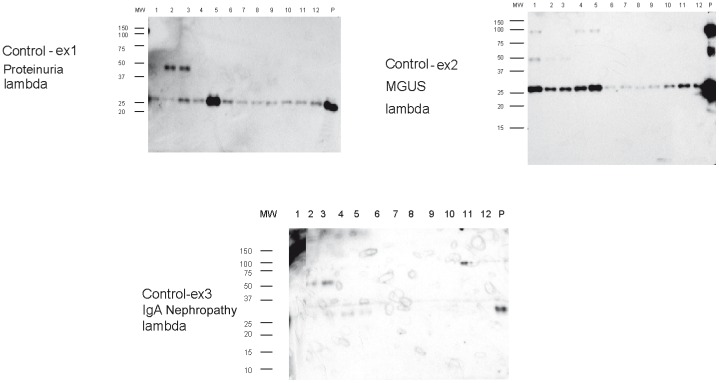
Western blot showing free light chain immuno-reactive proteins for controls. Control-ex1 is a patient with membranous glomerulonephritis. Control-ex2 is a patient with IgGλ+λ MGUS. Control-ex3 is a patient with IgA nephropathy. No free light chains were detected for control-ex4 (monoclonal λ protein) or for a healthy normal control (control-ex5), so no blots are shown for these two control samples.

#### Database searching

Tandem mass spectra were extracted and charge state deconvoluted by BioWorks version 3.2. Deisotoping was not performed. All MS/MS samples were analyzed using Mascot (Matrix Science, London, UK; version 2.2.04), Sequest (ThermoFinnigan, San Jose, CA; version 27, rev. 12) and X! Tandem (www.thegpm.org; version 2006. 09.15.3). X! Tandem was set up to search the SprotProt plus reverse database (Feb. 22, 2008, 699052 entries) assuming the digestion enzyme semiTrypsin. Sequest was set up to search the SprotProt plus reverse database (Feb. 22, 2008, 699052 entries) also assuming trypsin. Mascot was set up to search the SprotProt plus reverse database (Feb. 22, 2008, 699052 entries) assuming the digestion enzyme trypsin. Mascot and X! Tandem were searched with a fragment ion mass tolerance of 0.80 Da and a parent ion tolerance of 10.0 PPM. Sequest was searched with a fragment ion mass tolerance of 0.80 Da and a parent ion tolerance of 0.011 Da. Oxidation of methionine and iodoacetamide derivative of cysteine were specified in Mascot, Sequest and X! Tandem as variable modifications.

#### Criteria for protein identification

Scaffold (version Scaffold-2_00_02, Proteome Software Inc., Portland, OR) is used to validate MS/MS based peptide and protein identifications. Peptide identifications are accepted if they can be established at greater than 95.0% probability as specified by the Peptide Prophet algorithm [24]. Protein identifications are accepted if they can be established at greater than 95.0% probability and contain at least 2 identified peptides. Protein probabilities are assigned by the Protein Prophet algorithm [25]. Proteins that contained similar peptides and could not be differentiated based on MS/MS analysis alone were grouped to satisfy the principles of parsimony. In the case of AL-ex 1, the sequence obtained from the plasma cell cDNA was incorporated into the database for more accurate peptide identification.

## Results

Urine samples were collected from 8 AL amyloidosis patients (4 with monoclonal IgG or IgM protein and 4 with either λ or κ monoclonal protein), 5 MM patients and 5 control patients (Table 1). The control group consisted of 1 normal, 2 patients with monoclonal gammopathy with undetermined significance (MGUS) and 1 patient with IgA nephropathy and 1 membranous glomerulonephritis. The diagnosis for the AL amyloidosis was confirmed by renal biopsy using standard immunohistochemistry techniques. One MM patient had cast nephropathy confirmed on her renal biopsy. The other MM patients with normal renal function did not undergo a renal biopsy.

**Figure 4:**
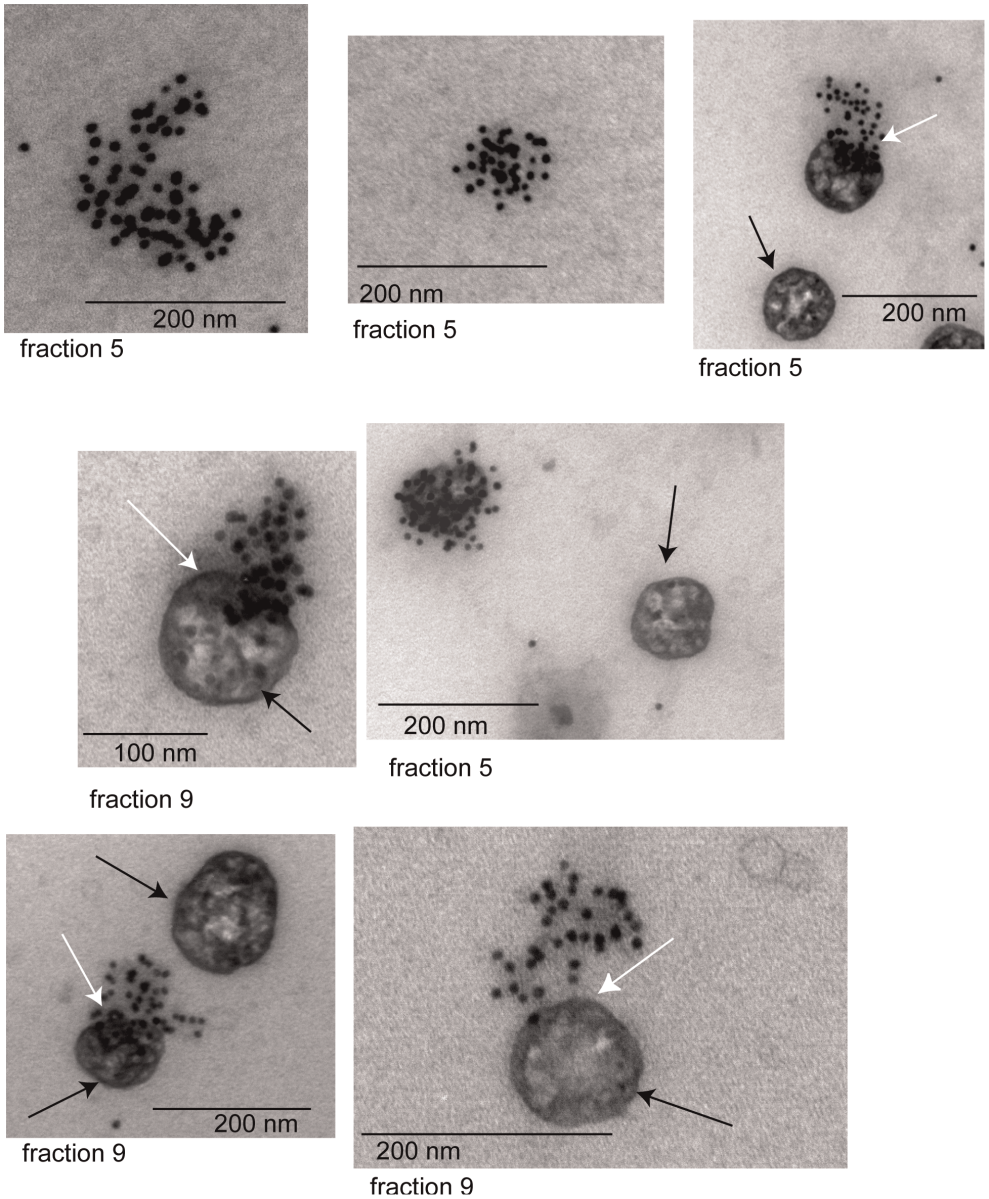
Collection of representative immuno-gold labeling images of patient AL-ex2 exosomes from fractions 5 and 9. Black arrows show MVB exosomes while white arrows point out the region on the exosome surface where accumulation of light chain labeling is present.

**Figure 5:**
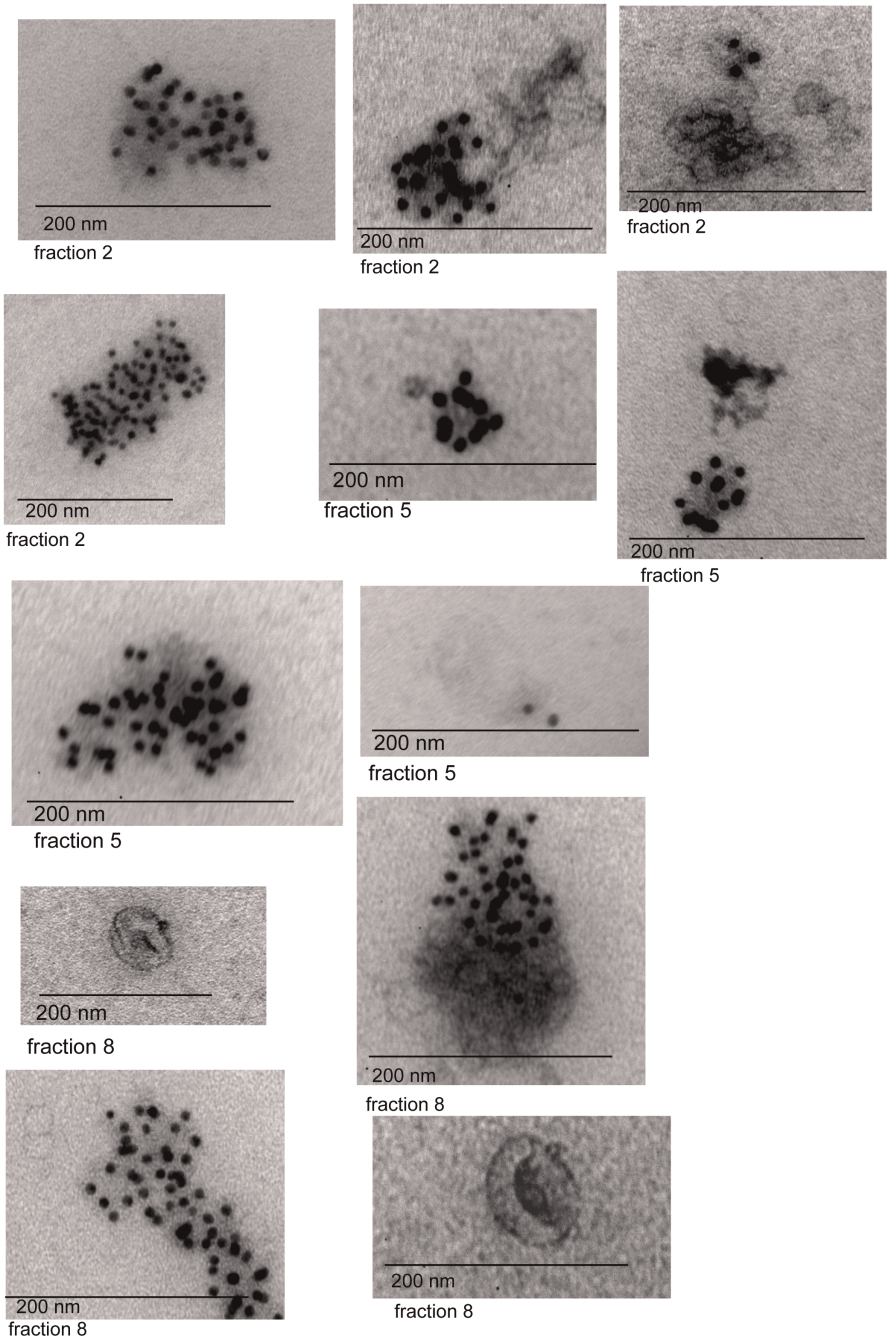
Collection of representative immuno-gold labeling images of MM patient exosomes, fractions 2, 5 and 9. There is a diversity of sizes and labeling within the fractions of this MM patient.

**Figure 6:**
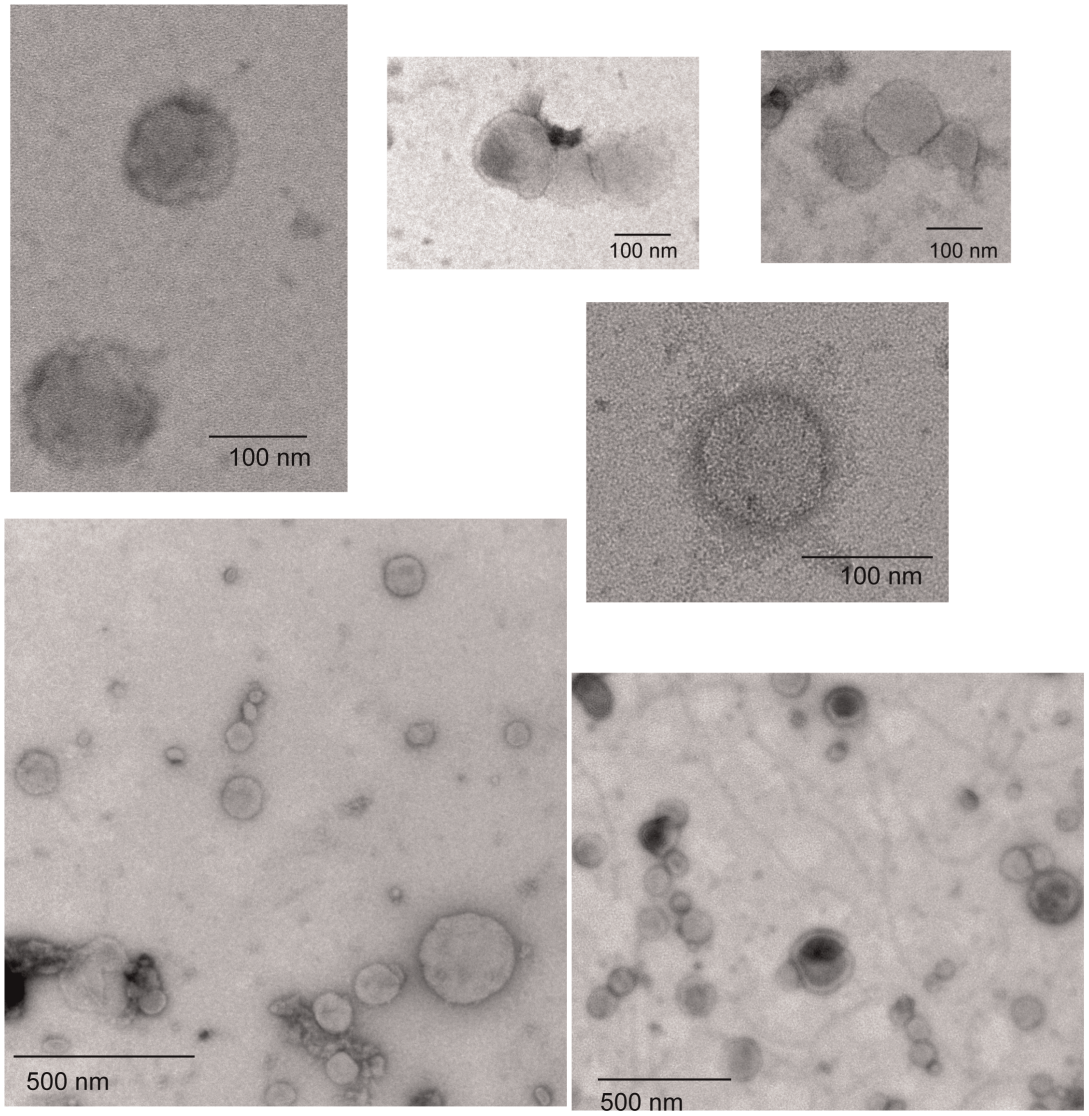
Collection of representative immuno-gold labeling images of exosomes from normal control, fraction 8. These images show the expected ‘deflated football’ structure for these vesicles.

Fresh gradient fractions containing different populations of exosomes were evaluated by Western blotting using free light chain and other antibodies. Fraction 5 and 6 were positive for podocin suggesting a glomerular origin (see figure S1). Previously published data had shown that fraction 2 corresponds to collecting ducts with immunoreactivity to aquaporin-2 and fractions 8–10 correspond to distal tubule exosomes with polycystin-1 immunoreactive protein [17]. The polycystin-1 immunoreactivity was confirmed in one patient in this study (data not shown).

The SDS-PAGE western blots showed that free light chains were present in exosomes from all AL, MM, one of the proteinuria patients, one of the MGUS patients, and the IgA nephropathy control (Figures 1, 2, 3). Free light chain bands were not identified in Control -ex4 and -ex5 (not shown). All samples showed that the majority of the exosome fractions had the expected 25 kDa band corresponding to the light chain monomer although the fractions containing the highest concentration of monoclonal light chain protein varied among the different diseases. The majority of AL amyloidosis samples showed the highest concentration of light chains in fraction 4–6. While the MM sample had the highest concentration in fraction 2, control-ex1 (membranous glomerulonephritis) has the highest concentration in fraction 5, while Control-ex2 (IgGλ MGUS) has high concentration in fractions 1 and 5. While the urine volume processed for each patient remained constant, the crude exosome pellet appeared to be different from sample to sample. It is also clear from figure 1 and 2 that the amount of immunoreactive protein found in the exosomes varies from patient to patient. Some exosome samples caused large perturbations in the gels (figure 2D, 2E, for example). These perturbations may be caused by specific properties associated with the exosomes from these particular patients.

Selective proteomics analysis was done analyzing slices from 1D SDS-PAGE gels representing all molecular weights. We found peptides corresponding to the monoclonal light chain in fraction 5 for AL-ex1 in all slices of the gel, while control-ex5 proteomic analysis only showed polyclonal peptides, mostly corresponding to the light chain constant domain in slices corresponding to a molecular weight of 25 kDa.

In AL exosome samples AL-ex1-AL-ex5, high molecular weight bands with immunoreactive for light chains were present in fractions 4–6. The maximum molecular weight detected was around 250 kDa corresponding to a light chain decamer. These high molecular weight bands were not detected in the SDS-PAGE western blots of the MM, MGUS or controls. For samples AL-ex6 and AL-ex7, these high molecular weight species were only found in the crude exosome preparations. Occasionally, dimers (∼50 kDa) were detected in patients with monoclonal lambda light chains which were expected since lambda can exist in dimeric state. In order to ensure that the light chain species we observed were not coming from the non-pathologic light chains, western blots were performed using antibodies against the alternative light chain (lambda for monoclonal kappa and kappa) for monoclonal lambda) and no immunoreactivity was detected in the selected samples tested, suggesting these bands were composed of the monoclonal light chains (figure S2). Another important control we ran was related to the high molecular weight bands that we observed in the AL amyloidosis samples. In order to confirm that these high molecular weight bands were composed exclusively of immunoglobulin light chains and not heavy chains, 4–15% polyacrylamide gradient gels were used on fraction 5 of selected AL samples and fraction 2 of the MM samples to better separate the high molecular weight bands. A western blot was then performed using a polyclonal antibody against human IgG. IgG was detected at 50 kDa (heavy chain only) and 150 kDa (intact immunoglobulin) in the AL patients (AL-ex1, AL-ex2 and AL-ex4) whose monoclonal protein contained a monoclonal IgG along with a monoclonal light chain (figure S3). No bands were detected at 250 kDa (which correspond to the light chain decamer). No IgG molecules were detected in AL-3 (used as the lambda control), in agreement with the finding that this patient only has monoclonal lambda light chain. Faint cross reactivity with AL-ex5 (IgM heavy chain) was detected, possibly due to the denaturing nature of the SDS-PAGE gels. The 5 MM samples in our study did not show any immunoreactivity with the IgG antibody by western blotting. No high molecular weight bands were detected in AL-ex8. This was a patient in complete hematologic and near complete renal response 4 years after a successful autologous stem cell transplantation.

Electron microscopy (EM) images of normal exosomes control-ex5 showed that the morphology of these exosomes is similar to the previously reported ‘deflated football’ with concave and convex surfaces (Figure 4). Immuno-gold EM analysis using the same free light chain antibodies used in the western blots showed light chains on the surface of AL and MM exosomes. Fraction 5 and fraction 9 were imaged for AL-ex2 exosomes. In fraction 5, where the SDS-resistant oligomeric species were found by western blot, a high concentration of light chains was present on the membrane of exosomes to the extent that it was difficult to find the membrane limits. In most cases, there was a homogeneous free light chain distribution on ‘deflated football’ AL exosomes (Figure 5).

EM images of fraction 2, 5 and 8 of MM-ex1 exosomes were taken for comparison. Fraction 2 had the highest concentration of free light chain as observed by western blotting (Figure 2). It correlated well with the amount of labeled free light chain observed with western blot. Fraction 5 was imaged to compare it with the fraction that contained oligomeric species in the AL exosomes. There was much less free light chain labeling for the MM-ex1 exosomes in this fraction compared to the AL exosomes, and no accumulation in a particular region of the exosome (Figure 6).

## Discussion

Urinary exosomes are becoming an important tool in the study of kidney disease [26]. In addition to their ability to enrich proteins of interest, they provide a glimpse into regions of the kidney that are otherwise difficult to access [27]. To the best of our knowledge, this is the first report of the use of urinary exosome in the study of patients with plasma cell dyscrasias, specifically patients with AL amyloidosis.

Our study showed that urinary exosomes from patients with AL amyloidosis and MM contain large amounts of free immunoglobulin light chains, although each patient shows differences in the amount of immunoreactive protein that appears to be consistent with the serum free light chain protein concentration (table 1). Controls samples from patients with MGUS, proteinuria and healthy individuals comparatively had little to no light chain present. The most interesting result however was the presence of SDS-resistant high molecular weight oligomers in the exosomes of the AL patients. These oligomers were found both in the crude exosomal preparations (AL-ex6 and AL-ex7) as well as in the fractions corresponding to the glomerular exosomes (AL-ex1 to AL-ex5). Even more exciting was the lack of high molecular weight oligomers in sample AL-ex8 who had a complete hematologic and renal response after treatment. It suggests that high molecular oligomers (up to a decamer) are found only in patients with active AL amyloidosis. No high molecular weight species were found in MM or MGUS urinary exosomes. These results are consistent with known pathophysiology where light chains from MM stays in its monomeric or dimeric forms while amyloidogenic light chains form higher molecular weight structures [2], [28]. In addition, the location of light chains in the exosome fractions correlated with the location on the site of kidney injury. Light chains were most prominent in fraction 5 and 6 of AL exosomes which stained positive for podocin indicating a glomerular origin. The exosomes fraction in MM showed that most of the light chains were in the distal tubular/collecting duct [28]. The control samples show a more diverse phenotype, possibly due to the fact that these samples came from patients with different conditions and therefore, the fraction with the largest amount of light chain reflected more of the unique condition the control patients suffer. Our results also confirmed that the high molecular bands we observed are made up of the pathologic light chain and not heavy chain.

Our data were consistent the previous experimental results on the trafficking of the glomerulopathic light chains in the kidney. Keeling et al. showed that amyloidogenic light chains underwent receptor mediated endocytosis and intracellular trafficking by mesangial cells [29]. The immuno-gold and Western blot studies clearly showed light chains were present on the surface of exosomes. Interestingly, Keeling and co-workers also found that amyloid fibrils were present extracellularly during their cell culture studies [29]. This suggests that the light chains are internalized into renal cells where the amyloid formation process begins and the amyloid fibrils are then shuttled out of the cells. The images of the large surface aggregates of light chains in AL exosomes may represent this process.

The results from this study suggest that urinary exosomes may be an excellent non-invasive tool for identifying patients with AL amyloidosis because high molecular weight light chain oligomers were found only in patients with AL. However, more studies are needed to confirm our observations. Particularly, it would be interesting to compare our results with patients with MIDD. Unfortunately, the rarity of MIDD precluded us from including these patients in this study. Currently, the diagnosis of AL requires a renal biopsy. While it is generally safe, it may not always be logistically feasible as patients may become coagulopathic from medications [30]. Isolation of exosomes with high molecular weight light chain oligomers would represent an enormous advantage which can be obtained at any time. From a scientific standpoint, the exosomes represent a miniature model of the extracellular and intracellular processes. The oligomeric light chain species may represent the initial steps of amyloidogenesis captured in the urinary exosomes. When combined with mass spectrometry and other proteomics techniques, urinary exosomes represent tremendous potential to increase our understanding of amyloidogenesis [27]. The potential of urinary exosomes in AL is tremendous and deserves further studies.

## Supporting Information

Figure S1 **Western blot using anti-podocin antibody identifying glomerular exosomes in fractions 5 and 6 of AL-ex1 (representative of all AL amyloidosis patients in this study).**
(TIF)

Figure S2 **Western blot using the uninvolved light chain for selected samples.** An anti-κ antibody was used for AL-ex1 through -ex4 while an anti-λ was used for AL-ex5.
(TIF)

Figure S3 **Western blot using a polyclonal anti-IgG antibody for selected samples.** Bands of ∼60 kDa, ∼120 kDa and ∼180 kDa were identified corresponding to monomeric and dimeric forms of the heavy chain and intact IgG in the control IgG sample. Bands were also identified in AL-ex1, ex2 and ex4 in whom the monoclonal protein included the heavy chain. In AL-ex4, who only had monoclonal λ protein, no band was identified. No immuno-reactive proteins were identified in the MM samples.
(TIF)

## References

[1] Bellotti V, Mangione P, Merlini G (2000). Review: immunoglobulin light chain amyloidosis–the archetype of structural and pathogenic variability.. J Struct Biol.

[2] Merlini G, Bellotti V (2003). Molecular mechanisms of amyloidosis.. N Engl J Med.

[3] Kyle RA, Gertz MA (1995). Primary systemic amyloidosis: clinical and laboratory features in 474 cases.. Seminars in Hematology.

[4] Comenzo RL (2009). How I treat amyloidosis.. Blood.

[5] Leung N, Rajkumar SV (2007). Renal manifestations of plasma cell disorders.. Am J Kidney Dis.

[6] Ivanyi B (1990). Frequency of light chain deposition nephropathy relative to renal amyloidosis and Bence Jones cast nephropathy in a necropsy study of patients with myeloma.. Archives of Pathology & Laboratory Medicine.

[7] Solomon A, Weiss DT, Kattine AA (1991). Nephrotoxic potential of Bence Jones proteins.. N Engl J Med.

[8] Leung N, Gertz MA, Zeldenrust SR, Rajkumar SV, Dispenzieri A (2008). Improvement of cast nephropathy with plasma exchange depends on the diagnosis and on reduction of serum free light chains.. Kidney Int.

[9] Pisitkun T, Shen RF, Knepper MA (2004). Identification and proteomic profiling of exosomes in human urine.. Proc Natl Acad Sci U S A.

[10] Vella LJ, Sharples RA, Nisbet RM, Cappai R, Hill AF (2008). The role of exosomes in the processing of proteins associated with neurodegenerative diseases.. Eur Biophys J.

[11] Porto-Carreiro I, Fevrier B, Paquet S, Vilette D, Raposo G (2005). Prions and exosomes: from PrPc trafficking to PrPsc propagation.. Blood Cells Mol Dis.

[12] Fevrier B, Vilette D, Archer F, Loew D, Faigle W (2004). Cells release prions in association with exosomes.. Proc Natl Acad Sci U S A.

[13] Rajendran L, Honsho M, Zahn TR, Keller P, Geiger KD (2006). Alzheimer's disease beta-amyloid peptides are released in association with exosomes.. Proc Natl Acad Sci U S A.

[14] Gomes C, Keller S, Altevogt P, Costa J (2007). Evidence for secretion of Cu,Zn superoxide dismutase via exosomes from a cell model of amyotrophic lateral sclerosis.. Neurosci Lett.

[15] Gonzales P, Pisitkun T, Knepper MA (2008). Urinary exosomes: is there a future?. Nephrol Dial Transplant.

[16] Zhou H, Pisitkun T, Aponte A, Yuen PS, Hoffert JD (2006). Exosomal Fetuin-A identified by proteomics: a novel urinary biomarker for detecting acute kidney injury.. Kidney Int.

[17] Hogan MC, Manganelli L, Woollard JR, Masyuk AI, Masyuk TV (2009). Characterization of PKD protein-positive exosome-like vesicles.. J Am Soc Nephrol.

[18] Gonzales PA, Pisitkun T, Hoffert JD, Tchapyjnikov D, Star RA (2009). Large-scale proteomics and phosphoproteomics of urinary exosomes.. J Am Soc Nephrol.

[19] Moon PG, Lee JE, You S, Kim TK, Cho JH (2011). Proteomic analysis of urinary exosomes from patients of early IgA nephropathy and thin basement membrane nephropathy.. Proteomics.

[20] Sikkink LA, Ramirez-Alvarado M (2008). Biochemical and aggregation analysis of Bence Jones proteins from different light chain diseases.. Amyloid.

[21] van Niel G, Raposo G, Candalh C, Boussac M, Hershberg R (2001). Intestinal epithelial cells secrete exosome-like vesicles.. Gastroenterology.

[22] Gharahdaghi F, Weinberg CR, Meagher DA, Imai BS, Mische SM (1999). Mass spectrometric identification of proteins from silver-stained polyacrylamide gel: a method for the removal of silver ions to enhance sensitivity.. Electrophoresis.

[23] Olsen JV, de Godoy LM, Li G, Macek B, Mortensen P (2005). Parts per million mass accuracy on an Orbitrap mass spectrometer via lock mass injection into a C-trap.. Mol Cell Proteomics.

[24] Keller A, Nesvizhskii AI, Kolker E, Aebersold R (2002). Empirical statistical model to estimate the accuracy of peptide identifications made by MS/MS and database search.. Anal Chem.

[25] Nesvizhskii AI, Keller A, Kolker E, Aebersold R (2003). A statistical model for identifying proteins by tandem mass spectrometry.. Anal Chem.

[26] Knepper MA, Pisitkun T (2007). Exosomes in urine: who would have thought…?. Kidney Int.

[27] Hoorn EJ, Pisitkun T, Zietse R, Gross P, Frokiaer J (2005). Prospects for urinary proteomics: exosomes as a source of urinary biomarkers.. Nephrology (Carlton).

[28] Winearls CG (1995). Acute myeloma kidney.. Kidney Int.

[29] Keeling J, Teng J, Herrera GA (2004). AL-amyloidosis and light-chain deposition disease light chains induce divergent phenotypic transformations of human mesangial cells.. Lab Invest.

[30] Soares SM, Fervenza FC, Lager DJ, Gertz MA, Cosio FG (2008). Bleeding complications after transcutaneous kidney biopsy in patients with systemic amyloidosis: single-center experience in 101 patients.. Am J Kidney Dis.

