# Supplementary figures and images for: Differences in Immunoglobulin Light Chain Species Found in Urinary Exosomes in Light Chain Amyloidosis (AL)
Source: PLoS One. 2012 Jun 18;7(6):e38061. doi: 10.1371/journal.pone.0038061 (PMC3377641; doi:10.1371/journal.pone.0038061)

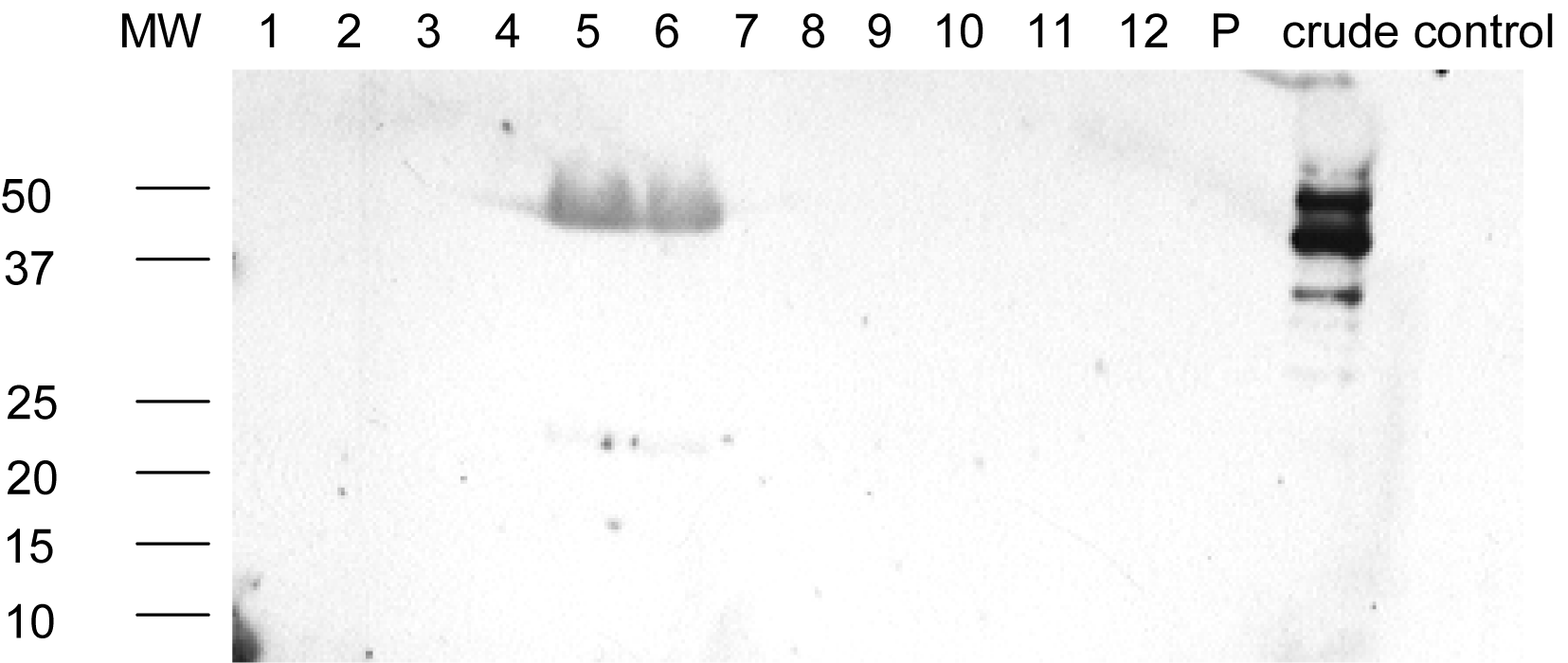

Supplement: Figure S1 — Western blot using anti-podocin antibody identifying glomerular exosomes in fractions 5 and 6 of AL-ex1 (representative of all AL amyloidosis patients in this study). (TIF) [file pone.0038061.s001.tif]

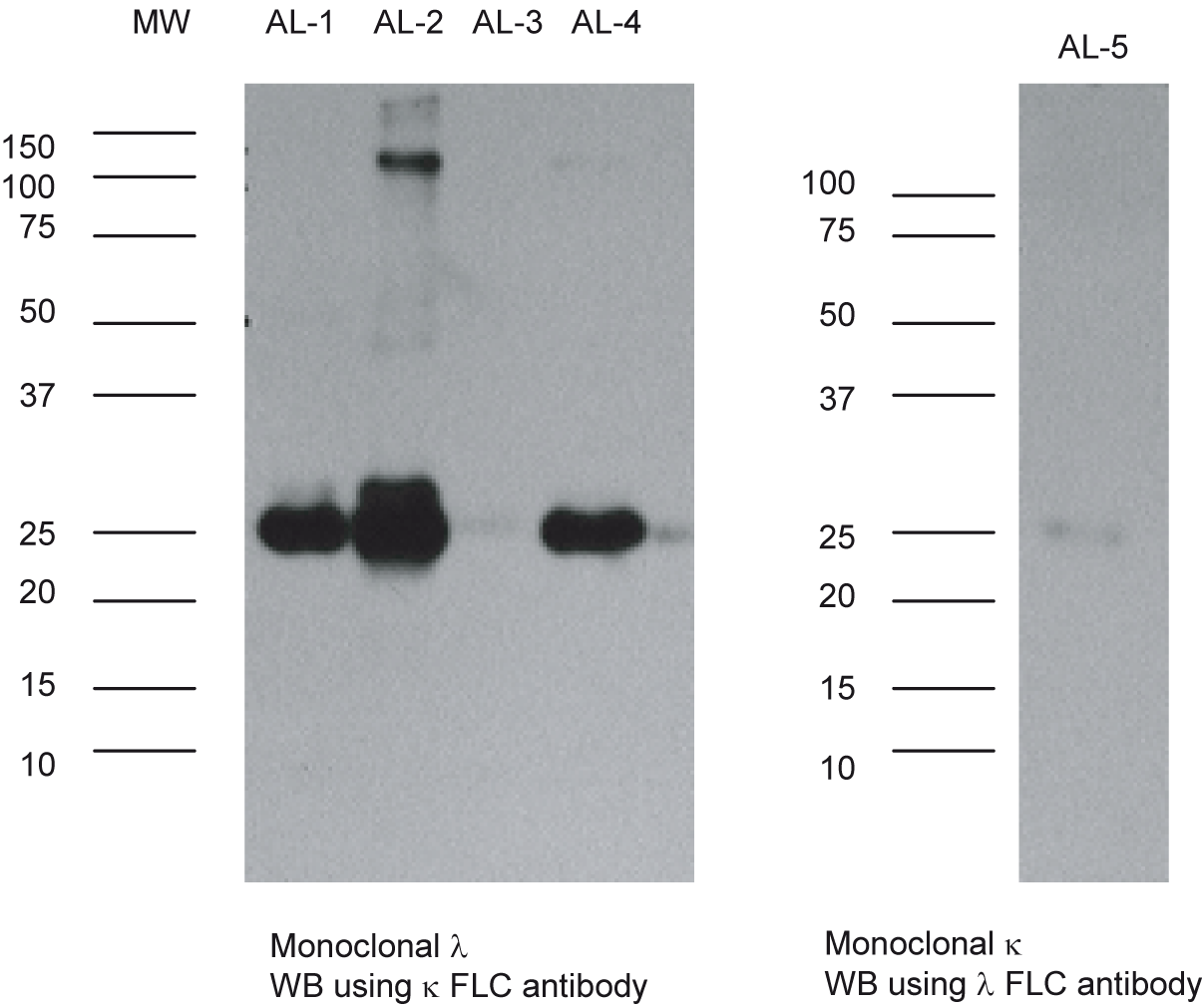

Supplement: Figure S2 — Western blot using the uninvolved light chain for selected samples. An anti-κ antibody was used for AL-ex1 through -ex4 while an anti-λ was used for AL-ex5. (TIF) [file pone.0038061.s002.tif]

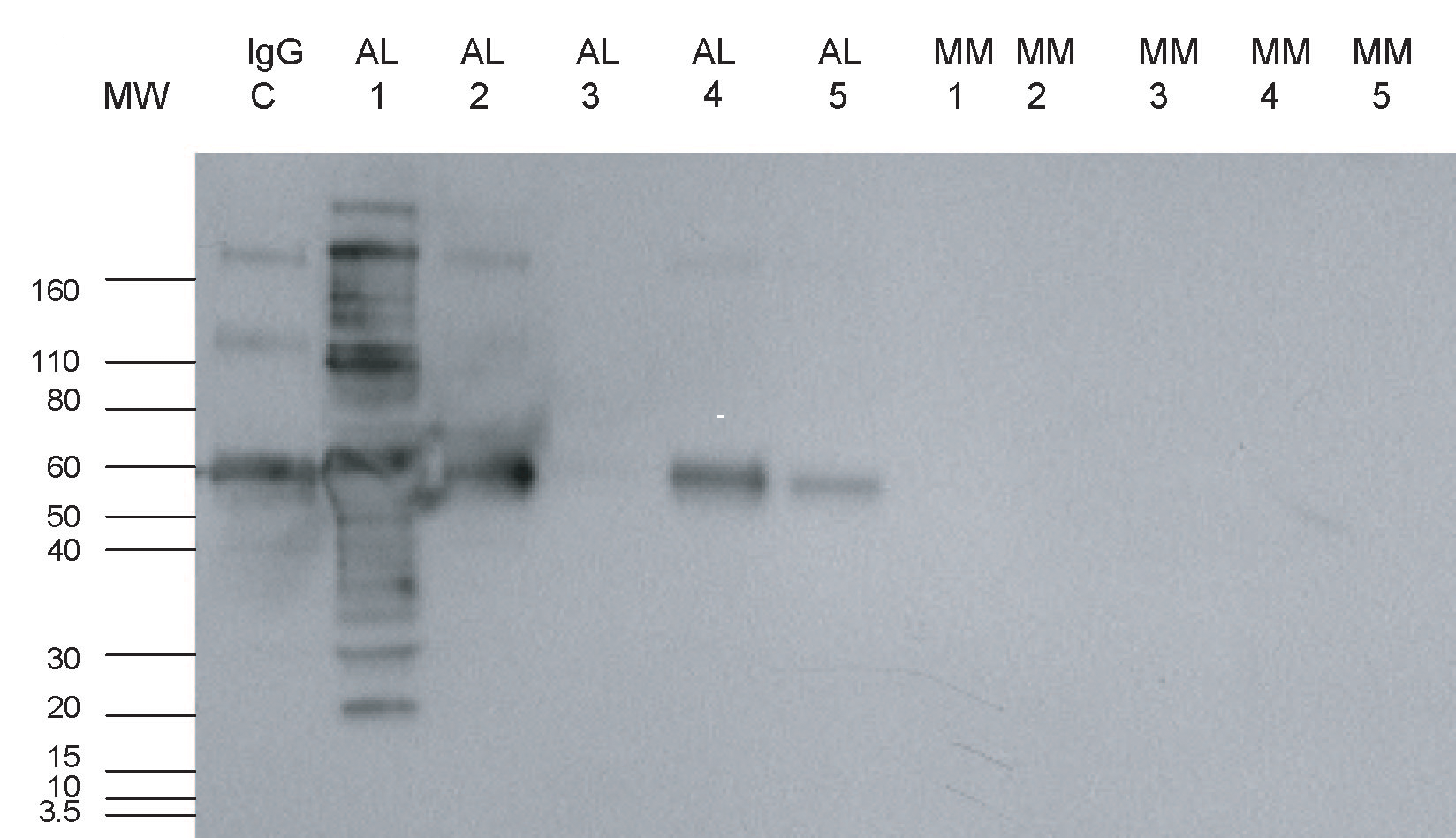

Supplement: Figure S3 — Western blot using a polyclonal anti-IgG antibody for selected samples. Bands of ∼60 kDa, ∼120 kDa and ∼180 kDa were identified corresponding to monomeric and dimeric forms of the heavy chain and intact IgG in the control IgG sample. Bands were also identified in AL-ex1, ex2 and ex4 in whom the monoclonal protein included the heavy chain. In AL-ex4, who only had monoclonal λ protein, no band was identified. No immuno-reactive proteins were identified in the MM samples. (TIF) [file pone.0038061.s003.tif]
